# The development of a knowledge test of depression and its treatment for patients suffering from non-psychotic depression: a psychometric assessment

**DOI:** 10.1186/1471-244X-9-56

**Published:** 2009-09-15

**Authors:** Adel Gabriel, Claudio Violato

**Affiliations:** 1 University of Calgary and Calgary Health region, 2000 Pegasus Rd NE, Calgary AB T2E 8K7, Canada; 2 Department Community Health Sciences, Faculty of Medicine, University of Calgary, 3330 Hospital Drive NW, Calgary AB T2N 4N1, Canada

## Abstract

**Background:**

To develop and psychometrically assess a multiple choice question (MCQ) instrument to test knowledge of depression and its treatments in patients suffering from depression.

**Methods:**

A total of 63 depressed patients and twelve psychiatric experts participated. Based on empirical evidence from an extensive review, theoretical knowledge and in consultations with experts, 27-item MCQ knowledge of depression and its treatment test was constructed. Data collected from the psychiatry experts were used to assess evidence of content validity for the instrument.

**Results:**

Cronbach's alpha of the instrument was 0.68, and there was an overall 87.8% agreement (items are highly relevant) between experts about the relevance of the MCQs to test patient knowledge on depression and its treatments. There was an overall satisfactory patients' performance on the MCQs with 78.7% correct answers. Results of an item analysis indicated that most items had adequate difficulties and discriminations.

**Conclusion:**

There was adequate reliability and evidence for content and convergent validity for the instrument. Future research should employ a lager and more heterogeneous sample from both psychiatrist and community samples, than did the present study. Meanwhile, the present study has resulted in psychometrically tested instruments for measuring knowledge of depression and its treatment of depressed patients.

## Background

Many people who have personal experience with depression cannot recognize it in vignettes, can't differentiate depression from normal sadness [1], their knowledge about its causes is distorted and over half of the subjects who have major depression (MD) do not seek treatment for the episode [2-4]. Moreover, only 40% consider antidepressants to be helpful [2], few recommend treatment from a counselor, telephone service or psychologist, and many consider a psychiatrist as harmful [3]. There is, however, emerging evidence to suggest that mental health literacy can be improved with educational interventions [5,6].

If the public's mental health literacy is not improved, public acceptance of evidence-based mental health care may be hindered. There is still much to be done to provide an empirical basis for evidence-based interventions to reduce misconceptions about mental illness and to improve attitudes toward people with mental illness [7,8].

Educational studies should include the appropriate measures to evaluate the effectiveness of psycho educational interventions. Some researchers have developed instruments to examine patient's knowledge of mood disorders and its treatments. Kronmüller et al., for example, developed the Knowledge about Depression and Mania Inventory (KDMI) in German, which demonstrated evidence for predictive prognostic validity [9,10]. Nonetheless, there are no strictly objective instruments (e.g., multiple choice questions - MCQ) readily available in English to assess knowledge of depression and its treatments in patients suffering from depression. There is, therefore, an urgent need to develop materials and methods to teach depressed patients, and reliable and valid instruments to measure and assess patients' knowledge of depression.

The major purpose of the present study was to develop and psychometrically assess an MCQ instrument to measure patients' knowledge of depression. A number of themes about patients' and the public's lack of knowledge of depression emerge and are summarized below.

### Recognition of Depression and Helpful Professionals

Many people are not able to identify depression correctly in community surveys or structured interviews of both adolescents and adults [2-4,11-13]. In these studies, respondents were also misinformed about the causes of depression, were not able to differentiate major depression from normal sadness, and were unlikely to seek professional help for depression [12-17]. In a vignette depicting a depressed person, for example, only 39% of respondents (n = 1 010) correctly identified the case as depression. Moreover, only 51% rated a psychiatrist as helpful from a list of various professionals that could be either helpful or harmful for the person described in the vignette [11]. Many standard psychiatric treatments (antidepressants, antipsychotics, electroconvulsive therapy, and admission to a psychiatric ward) were more often rated as harmful than helpful, and some nonstandard treatments (increased physical or social activity, relaxation and stress management, reading about people with similar problems) were rated as more helpful [11].

### Knowledge about the Causes of Depression

There are many imprecise beliefs about the causes of depression among both patients and the public, which appear to influence the perceptions of the effectiveness of treatments. In a number of studies [14-16] there is evidence to suggest that, especially among poorly educated people, there is an enduring belief system that depression is primarily caused by psychosocial stresses such as occupational and family stressors or by weakness of character or losing self-control. In the study by Lauber, Falcato, Nordt and Rossler, for example, only 14.1% of participants (n = 873) attributed symptoms to depression when presented with a vignette depicting a man with depressive symptoms, while more than half considered family difficulties, occupational difficulties, or other traumatic factors as the main causes for the symptoms [15]. Poor knowledge of the causes of depression and its biological aspects is widespread in patients with depression [17-19].

### Knowledge of Depression and its Causes Influence Treatment Choices

A number of studies have shown that imprecise knowledge of depression and its causes negatively influence the decision of treatment choices. In a community survey (n = 3 010), for example, although people with personal experience of depression viewed depression as more disabling than other medical conditions, 40% of those with major depression considered antidepressants harmful [2,14]. Psychiatrists are frequently viewed as not very helpful for depressed patients [2,3,14,20-23]. Many people with depression frequently turn to the lay support system first, followed by the family physician if the former fails to help [24]. Some studies have found that correct recognition of depression and attribution to biological causes is associated with a positive attitude toward psychopharmacology [11,12,17,18,25,26].

### Poor Knowledge of and Negative Attitudes to Depression Influence the type of Help-Seeking

It was reported that 55% of subjects who fulfilled the Research Diagnostic Criteria of Major Depression did not seek help [4]. The non-help seekers did not consider the episode serious or recognize it at as an illness and believed that they could handle the episode themselves. On the other hand, those who sought help felt that their experience of the episode was too painful, lasted too long, and disrupted their interpersonal and role functioning [4]. A number of studies have shown that the most frequently endorsed reasons for depressed people delaying or not seeking professional help or treatment was related to lack of knowledge about mental illness and available treatments [4,12,27,28]. People who have poor knowledge of depression are less likely to recommend treatment from a counselor, psychologist, or a psychiatrist than those who had better knowledge - some with poor knowledge consider psychiatrists to be harmful [3,24].

Most of the community and patient studies have relied on vignettes, anecdotes or case studies for assessing respondents' knowledge of depression and other mental health conditions. There are no studies that used reliable and valid instruments to objectively and comprehensively test the patient's knowledge of depression such as multiple choice question (MCQ) instruments. Vignettes were the most commonly used methods in various studies and surveys to assess recognition of depression by both patients [23-26] and the public [29-32]. There is, therefore, an urgent need for reliable and valid instruments to assess knowledge of depression by patients and the general public. Accordingly, the major purpose of the present study was to develop and psychometrically assess an instrument for measuring knowledge of patients suffering from depression.

## Method

### Participants

#### Patients

A total of 63 consenting, stable depressed patients and twelve psychiatric expert volunteers participated. The 63 patients, both men and women, ranged from 18 to 65 years of age (mean = 43 years). All participants were treated as outpatients following referrals by their family physicians. The "Mini-International Neuropsychiatric Interview" (MINI screen 2001-2005) was used to confirm the diagnosis of major depressive or dysthymic episodes [33]. Patients were included if they had at least one episode of major depression, dysthymia, or bipolar depression. All patients were clinically stable (i.e. not acutely depressed or exhibiting suicidal ideation, and those who scored less than 4 on the Hamilton Rating Scale for Depression (HAM-7) [34].

All patients were on antidepressant medication, and all had seen their clinicians on at least two occasions for standard treatment and standard psycho-education as a part of standard clinical care prior to recruitment. Patients with chronic or recent alcohol and illicit drug abuse, patients suffering from psychotic symptoms, and patients suffering from all degrees of mental handicap, were excluded from the study.

#### Psychiatry Experts

Both male and female experts in mood disorders were invited to participate in the present study (n = 12, female/male = 2/10, mean age = 52 years; SD = 11.6, and mean years of experience as independent consultants = 22; SD = 12.5). There were nine at the rank of professor, two at associate professor, and one at assistant professor. Each expert reviewed and provided comments on the relevance of the instrument to be developed before testing the instruments with patients suffering from depression. Three experts were invited for an informal panel discussion of the instrument, and reviewing the individual items in depth. Each of the remaining nine experts was invited to formally rate each item for its relevancy in testing depression knowledge and its treatment on a five point Likert scale.

The conjoint scientific and ethics board of the University of Calgary granted approval for the study.

### Procedures

The design involved the development and the psychometric assessment. A table of specification (Table 1), with the initial items was created to guide the question construction (items = 27), on three levels of cognitive outcomes: knowledge, comprehension, and application [35]. The initial items of the table of specification were developed based on empirical evidence from an extensive review of literature, theoretical knowledge, and in consultations with national and international psychiatry experts. The instrument items were divided into the following five subscales: Definition (5 items), risk of relapse (2 items), etiology (2 items), presentation and symptoms (6 items), and biological and psychological treatments (12 items). The MCQ items (Appendix A) were written following basic rules for item construction so as to avoid common technical item flaws [36,37]. A volunteer panel of experts met on three occasions to review the items for the following: 1) appropriateness of difficulty and relevancy for patients as examinees, 2) concise, clear language at the appropriate level (Grade 9) and was as much as possible without medical or psychiatric jargon, 3) each requires patient knowledge to be demonstrated in a specific area of depression or its treatment, and 4) at least three experts agreed on the correct answer for each question.

**Table 1:** Table of Specifications and Taxonomy of the Cognitive Objectives

**Knowledge Objectives**	**KNO^¶^**	**COM^±^**	**APP^†^**	**Total**
Definition The ability to understand that depression is not a weakness of the character, but a medical disorder.		^††^Q1		1

The Size of the Problem (Epidemiological facts) Prevalence of depression Having the correct knowledge about the life time chances of becoming depressed approximately	Q3			1

The risk of suicide Awareness of the serious facts about the rates of suicide associated with depression	Q2			1

Age of Onset Recognizing that depression can start in childhood	Q4			1

Sex differences Recognizing that depression is more common in women than men	Q5			1

Relapse risks of, and triggering factors Knowledge of the chances of relapse rates after remission Understanding that sopping antidepressants after recovery may lead to relapse	Q6	Q7		2

Etiology The knowledge that depression could be predisposing or triggered by multiple Biological and Psycho-social factors	Q8	Q9		2

Clinical Presentations				

Distinction from normal sadness Awareness that occasional sadness may not be an indication for clinical depressive disorder. The comprehension that suffering from depression may need more than helping oneself Recognizing that an important difference from normal sadness that depression may last much longer, without treatment	Q10	Q11		2

To recognize the common symptoms of clinical depression,	Q12			4
Cognitive deficits	Q13			
Inability to make decisions	Q14			
Abnormal thought content, cognitive abnormalities, and Poor energy	Q15			

Knowledge of Biological treatments (antidepressants)				

Knowledge of the delayed onset of the action of antidepressants	Q16			1

Ability to act appropriately to failed response to antidepressants			Q17 Q21	2

Ability to act and respond appropriately to positive response to antidepressants			Q23	1

Ability to understand the need for maintenance treatment		Q25		1

Knowledge of different kinds of treatments	Q18			1

Knowledge of the magnitude of therapeutic efficacy of treatments	Q20			1

Knowledge of common side-effects	Q19 Q24			2

Predicting success of treatment with antidepressants	Q26			1

ECT knowledge	Q22			1

Psychological treatments	Q27			1

Total	20	4	3	27

^¶^KNO: **Knowledge **^±^COM: **Comprehension **^†^APP: **Application **^††^Q: **Question**

The remaining nine experts were asked to rate the relevance of each MCQ in sampling patient knowledge of depression and its treatment on a 5-point Likert scale (from 1 = irrelevant to 5 = highly relevant).

### Data Collection and Analysis

Responses from psychiatry experts were used to provide evidence for face and content validity for the instrument, while patient responses and patient performances on the instrument were utilized to provide evidence for internal consistency reliability and convergent validity as presented in correlation analysis, and factor analysis. For the patients, each item on the MCQ test was scored correct (1) or incorrect (0) and then summed for a total score.

## Results

The demographics of the patients are described in Table 2. Most of the sample were women (65%), Caucasian (86%) and had suffered from depression for a mean number of 8.9 years (SD = 6.3).

**Table 2:** Demographics of Participating Patients (n = 63)

**Non-continuous variables**	**Frequency**	**Percentage %**
Sex		
Men/Women	22/41	35/65

**Diagnosis**		
Major Depression	44	70
Bipolar Depression	14	22
Dysthymia	5	8

Marital Status		
Single	51	24
Married	33	52
Divorced	12	19
Separated	3	5

Ethnicity		
Caucasian	54	86
Non-Caucasian	9	14

Occupation		
Professional	14	22
Entrepreneur	7	11
Skilled	26	41
Non-skilled	16	26

**Continuous Variables**	**Min/max**	**Mean ± SD**

Patient age	19/65	43 ± 11.3

Duration of depression (years)	1/25	8.9 ± 6.3

Duration of the most recent episode (months)	1/20	6.8 ± 3.8

Times of visits over last 6 months	1/10	5 ± 2.4

HAM-D 7 score at enrolment	0/4	2.38 ± 1

The mean score of performance on the MCQs was 21 (SD = 3.1) with a range 12 - 26. The internal consistency reliability (Cronbach's alpha) was 0.68 for the 27 items on the MCQ. There were no significant differences between males and females, in marital status, ethnicity or any differences among occupational groups in the performance on the MCQ. Nor were there significant correlations between the duration of depression and the number of visits to see a psychiatrist, or a counselor, and the performance on the MCQ scores.

### Item Analyses

For the purpose of item analyses (Table 3), patient performance was categorized into the following groups:

**Table 3:** MCQ Distribution and Item Analysis of the Knowledge MCQ

**Item**	**Percentage of responses for each item**		**Percentage of High and Low Students Responding to Each Option**								**Total**		
						
			**A**		**B**		**C**		**D**				
	**Correct**	**Wrong**	**High**	**Low**	**High**	**Low**	**High**	**Low**	**High**	**Low**	**K**	**P**	**D**

1	87	13	4	9	95	45	0	36	0	9	B	0.87	0.40

2	51	49	12	18	58	54	16	18	12	9	B	0.51	0.13

3	60	94	4	0	12	81	83	9	0	9	C	0.60	0.80

4	87	13	0	9	91	72	4	18	4	0	B	0.87	0.20

5	89	11	0	0	95	72	4	27	0	0	B	0.89	0.27

6	65	35	0	0	4	27	4	36	91	36	D	0.65	0.47

7	92	8	0	0	0	9	0	18	100	72	D	0.92	0.27

8	94	6	0	9	0	9	0	9	100	72	D	0.94	0.20

9	84	16	0	9	100	45	0	18	0	27	B	0.84	0.47

10	46	54	41	54	0	18	0	18	58	9	D	0.46	0.67

11	54	46	62	36	37	54	0	9	0	0	A	0.54	0.33

12	94	6	0	9	0	9	0	0	100	81	D	0.94	0.13

13	67	33	20	9	0	0	79	63	0	27	C	0.67	0.33

14	94	6	0	0	0	0	0	18	100	81	D	0.94	0.13

15	91	9.5	0	0	95	81	4	9	0	9	B	0.91	0.13

16	87	13	95	54	4	27	0	0	0	18	A	0.87	0.33

17	95	5	4	0	95	90	0	0	0	9	B	0.95	0.00

18	91	9.5	0	0	0	9	0	18	100	72	D	0.91	0.20

19	67	33	8	54	4	0	4	18	83	27	D	0.67	0.67

20	87	13	4	9	0	0	0	18	95	72	D	0.87	0.13

21	91	9	100	100	0	0	0	0	0	0	A	0.91	0.07

22	33	67	8	18	4	27	50	9	37	45	C	0.33	0.40

23	98	2	0	0	100	100	0	0	0	0	B	0.98	0.00

24	38	62	0	9	70	18	12	36	16	36	B	0.38	0.53

25	92	8	0	9	0	9	0	9	100	72	D	0.92	0.20

26	89	11	4	18	0	0	0	18	95	63	D	0.89	0.20

27	87	13	0	9	0	0	100	63	0	27	C	0.87	0.27

K, Key (correct) Response; P, Difficulty Index; D, Discrimination Value

A, B, C, & D: Response choices to each possible answer for each item in the High and Low performer groups.

Discrimination values are identified as follows;

0. 5 -- 1.0 High discrimination (items n = 4)

0.3 -- 0.5 Moderate discrimination (items n = 7)

0.1-- 0.3 Some discrimination (items n = 13)

< 0.1 Poor discrimination (items n = 3)

1. Low performers, with a score range of 12-16 (n = 6)

2. Average performers, with a score range of 17-21 (n = 22)

3. High performers, with a score range of 22-26 (n = 35)

In Table 3, K refers to the Keyed (correct) response, P is item difficulty (the percentage of patients who answered this item correctly), and D is the discrimination index of the item (how well this item distinguished between the poor and the high performers on the MCQ test):



*P*_*h *_represents the proportion of patients in the High performance group who answered the item correctly, *P*_*l *_represents the proportion of patients in the Low performance group who answered the item correctly, and *n *is the number of all patients who tried this item (Table 3). There were no significant differences among the high and the poor knowledge-performers in the three groups for age, durations of illness, the duration of the current episode, and the number of visits with a psychiatrist over the last six months.

### Experts' Responses

The expert rating of the relevance of each item for meeting the objective of measuring and testing patient knowledge of depression is summarized in Table 4. Items were rated as follows: 1 as irrelevant, 2 as slightly relevant, 3 as moderately relevant, 4 as significantly relevant, and 5 as highly relevant. There were no significant differences in ratings among experts based on their length of experience. There was an overall agreement (88%) among experts about the relevance of the MCQs to test patient knowledge on depression and its treatments. The majority of the items were rated as highly or significantly relevant (mean = 4.4, SD = 0.67, range = 1-4).

**Table 4:** Expert Agreement, Patient Responses and the Reliability of the MCQ Knowledge Subscales

**Knowledge Subscales**	**Items**	**Expert's Agreement (%)**	**Reliability Cronbach's**	**Patients' Correct Responses (%)**
1. Definition, the size of the problem	5	80	0.11	75

2. Risks of relapse	2	97	0.32	75

3. Etiology, causes, and triggers of depression	2	91	0.70	86

4. Presentation and symptoms	6	86	0.44	77

5. Biological and psychological treatments	12	86	0.61	81

Overall Content Validity & Reliability	27	88	0.68	78.8

There was significant positive relationship (r = 0.35, *p *< 0.01; r = 0.33, *p *< 0.05), between having the necessary knowledge about the risks of relapse (subscale #2) and being aware of the symptoms of depression (subscale #4), on the one hand, and having knowledge of different biological and psychological treatments (subscale #5), respectively. It could be concluded that when patients understand the causes of depression, they will be able to think of treatment options more rationally. There was also positive correlation (r = 0.30, *p *< 0.05; r = 0.27, *p *< 0.05) between subscale 5 'understanding biological and psychological treatments', and subscale 3' knowledge of etiology and triggers of depression, and subscale 4, 'knowledge of symptoms' respectively.

#### Reliability

The total test had an internal consistency of 0.68 and although internal consistency for subscales #3, #4 and # 5 were 0.70, 0.44, and 0.61, subscale #1 (items = 5) and subscale #2 (items = 2) have a much lower internal consistency of 0.11 and 0.32. Some of the items in these two subscales (items = 7), however, have good discriminating values that ranged from 0.40 to 0.80 in three out of the seven items. The low reliability is due to the poor variability among the individual scores on the items within these subscales.

#### Factor Analysis

Principal component analysis applied on the 27 MCQs item collected from the psychiatric out-patient setting revealed seven principal components that explain 57.6% of the variance related to patient's responses on knowledge about depression and its treatments (Table 5).

**Table 5:** Rotated Factor Matrix for the MCQ Instrument*

	**Component Loadings**						
**Items**	**C1**	**C2**	**C3**	**C4**	**C5**	**C6**	**C7**

1. Which of the following statements about the speed of response to the treatment with antidepressants is FALSE?	.75						

2. Which of the following about sex differences in depression is true?	.75						

3. All of the following are recognized symptoms of depression EXCEPT:	.73						

4. Which of the following is true about the age of onset of depression?	.69						

5. All of the following are typical of patients suffering from clinical depression EXCEPT:	.64		.43				

6. What are the lifetime chances of becoming clinically depressed?		.75					

7. Which is FALSE about the response to treatment with antidepressants?		.63					

8. What factors may trigger the onset of clinical depression?		.61					

9. Depression may be triggered by all the following EXCEPT		.50					

10. Which of the following statements about clinical depression is False?		.49					

11. If medication does not improve depressive symptoms, one should:			.78				

12. Which is FALSE about the effectiveness of antidepressant medications?			.75				

13. Which of the following behavior is associated with poor outcome?			.67				

14. Which is NOT a common symptom of clinical depression?			.44				

15. Which of the following is NOT a symptom of clinical depression?			.42				

16. Psychotherapy can help many people with depression. Which of the following statements about psychotherapy is FALSE?				.87			

17. Which is FALSE about selecting the right antidepressant, for someone with depression?		.44		.68			

18. Which is NOT a recognized treatment for clinical depression?					.73		

19. The following symptoms are indications of clinical depression EXCEPT:					.57		

20. Which of the following is FALSE about the relapse of clinical depression?					.47		

21. What should one do if one's first antidepressant medication fails?						.46	

22. What is the risk of dying by suicide among depressed patients?						.59	

23. Which is NOT true about the differences between depression and a passing blue mood?						.57	

24. If one feels better during the course of treatment, one should						.48	

25. Which is NOT a common occurrence during treatment with antidepressants?							.82

26. Which is FALSE about Electric Convulsive Therapy (ECT) for treating clinical depression?							.49

27. Which is NOT a common side effect of antidepressant drugs?							.45

**Internal Consistency Reliability (Alpha)**	.79	.33	.60	.64	.46	.13	.51

**Percent of Observed Variance**	15.7	9.5	8.2	7	6.2	5.4	5.2

*Principal Components Extraction, Varimax Rotation with Kaiser Normalization, Rotation Converged in eight iterations

### Component 1: The Presenting Profile

This component consists of 5 items, has an internal consistency of 0.79 and explains 15.7% of the observed variance. This component refers to the knowledge of the antidepressants and their delayed action, especially in patients with significant symptoms, such as melancholic features and cognitive impairments.

### Component 2: Etiology

This component consists of 6 items, has an internal consistency of 0.33, and explains 9.5% of the observed variance. This component refers to the fact that despite that the lifetime chances of becoming clinically depressed is high and that there are many life stresses that can trigger depression, there is hope for recovery with treatment using antidepressants.

### Component 3: Symptoms' Response to Treatments

This component consists of 6 items, has an internal consistency of 0.60, and explains 8.2% of the observed variance. This component refers to the knowledge about the expected patient's behavior in order to achieve clinical response and improvements in symptoms, and better prognosis.

### Component 4: Psychotherapy

This component consists of 2 items, has an internal consistency of 0.64, and explains 7% of the observed variance. It refers to correct knowledge about psychotherapy and the challenges associated with selecting the right antidepressant for a particular patient.

### Component 5: Subtle Symptoms of Relapse

This component consists of 2 items, has an internal consistency of 0.46, and explains 6.2% of the observed variance. This component refers to the knowledge about the risk factors and symptoms associated with relapse of the illness.

### Component 6: Challenges to Adherence

This component consists of 4 items, has an internal consistency of 0.13, and explains 5.4% of the observed variance. This component refers to the patients' ability to recognize the normal from the abnormal mood states and what is expected from them to do when they feel depressed or when antidepressants fail.

### Component 7: Biological Treatments and its Side-effects

This component consists of 3 items, has an internal consistency of 0.51, and explains 5.2% of the observed variance. It refers to the awareness of the common side-effects of antidepressants and the efficacy of electro-convulsive treatment.

## Discussion

The main findings of the present study are: 1) psychiatry experts have a high agreement on the content of an MCQ test of depression and on the relevance of specific items thereby adducing evidence of content validity, 2) Cronbach's alpha of the instrument was 0.68 indicating adequate reliability, 3) item analysis indicated that most of the items were working well producing appropriate difficulties, discrimination, and distracter effectiveness, and 4) the patients performed, overall reasonably well on the MCQ test.

While the total test had adequate reliability, two subscales did not. Reliability of these subscales can be improved by decreasing the difficulty of some items (e.g. item # 3) for which performance was poor in both the high and the low groups and to increase the difficulty of some of the very easy items (items #4 and #5), thus increasing the variance and leading to improve internal consistency reliability. Future research might also focus on test-retest reliability.

### Evidence for Content Validity

This is supported by two main factors. First, the MCQ test was initially developed based on empirical evidence from extensive literature review, and from consultations with experts in the field of depression. Second, from assessment of the instrument, there was 88% overall agreement among experts on the relevancy of its contents to measure patient knowledge of depression and its treatments with the means very high (4.4) for highly relevant.

### Evidence for Convergent Validity

Evidence of convergent validity exists when there are positive correlations between subscales as theoretically expected. The intercorrelation between risks of relapse (subscale #2) and awareness of the symptoms of depression (subscale #4), and having knowledge of different biological and psychological treatments (subscale #5), provide evidence of convergent validity. Similarly, the positive correlations subscale 5 'understanding biological and psychological treatments' and subscale 3' knowledge of etiology and triggers of depression, and subscale 4, 'knowledge of symptoms' support convergent validity. Also there were positive correlations between the different subscales.

### Patients' Performance on the MCQ Instrument

The majority of patients did generally well on the test (mean of the test = 78.8%) of patients answered items correctly). Fully 77% were able to answer questions about recognizing the symptoms of depression correctly, and 86% answered questions about causes of depression correctly. This is in contrast to a number of studies showing that less than 50% of community participants were able to differentiate depression from normal sadness [13,16]. Other population based surveys have similarly demonstrated that most respondents attribute the cause of depression to family or partnership difficulties [15]. Our present findings are in contrast to published findings that patients and members of the public failed to recognize depression in vignettes [2,4,7].

The evidence from previous research indicates that respondents have poor knowledge about, and negative attitudes to antidepressants. Medical treatments for depression were proposed by a minority of respondents, and only those who are were able to recognize depression in a vignette [17,20,22]. In the present study, however, 80% of patients gave correct answers to questions about the treatments especially antidepressants.

Finally, patients in the present study correctly answered 81% of questions about different treatments of depression. The high performance for the majority of patients on this instrument could reflect having developed and administered items, which were relatively easy, to a highly knowledgeable patient sample. Item analysis seems to support this conclusion.

### Items' Discriminating Power

There were eleven highly discriminating items (D = 0.3 - 1), thirteen slightly discriminating items (D = 0.1 - 0.3), and three poorly discriminating (17, 21, and 23; D < 0.1) items. Some of the items, appeared very easy, and their answers were obvious, thus leading to poor discrimination between the high- and the low-performing patients (i.e., MCQ s # 17, 21, 23). For example, #17 stem reads, "If my medication does not improve depressive symptoms, I should ...". To the majority of patients the correct answer was obvious (B), "Talk to a health-care professional." Reviewing the distracters of this item, option A, "Stop taking all medication" requires review as it is obviously inappropriate and undesirable and very easily excluded. Option D, "Ask friends about what to do" appears as a good distracter, in that it shows differences between the high and the low performing groups. None of the high performers selected this distracter while 9 of the low performers selected it. Also, the distracters C and D in items 21 and 23 did not show any discrimination between the high and the low performing groups. Modifying the distracters to increase the difficulty level of these items can make these items more discriminating. Alternatively, changing the distracters to increase the difficulty level of these questions can make these items more discriminating.

There were no significant differences among the high and the poor knowledge-performers in the three groups with respect to age distribution, durations of illness, and the duration of the current episode, and the number of visits with a psychiatrist over the last six months.

There are limitations of the present study. The sample of patients was not large, was homogenous and all patients were recruited from the authors' practice. Future research should include larger, more heterogeneous samples from various community clinics. Also the instrument contains some very easy items leading to poor discriminating power for these items and the comparative lack of difficult items. In future research the instrument might include items assessing other treatment possibilities in depression such as the preference for psychotherapy for many people who are clinically depressed.

## Conclusion

A reliable and comprehensive MCQ instrument (items = 27), to measure educational domains of knowledge, in patients suffering from depression was developed. There is evidence for content and convergent validity for this instrument as well as internal consistency reliability. In future research the instrument should be administered to a lager sample, after reviewing the poor items, and removing items numbers which proved poorly discriminating.

## Competing interests

The authors declare that they have no competing interests.

## Authors' contributions

This study is based on an MSc thesis of AG that was supervised by CV. Both authors conceived of the study and participated in its design and coordination. AG administered the instruments and collected the data. CV directed and oversaw the statistical analysis which was conducted by both authors. Both authors participated in the writing and revision and approved the final manuscript.

## Appendix A: Knowledge of depression MCQ Test

**Instructions**: Circle the best answer for each question

1. Which of the following statements about clinical depression is FALSE?

a. It is a medical disorder.

b. It is a weakness of character.

c. It is a common psychiatric disorder.

d. It affects both males and females.

2. What is the risk of death by suicide among depressed patients?

a. The risk is very minimal.

b. The risk is between 15% and 50%.

c. The risk is below 15%.

d. The risk is above 50%.

3. What are the lifetime chances of becoming clinically depressed?

a. One in 1000

b. One in 50

c. One in 3

d. One in 1

4. Which of the following is TRUE about the age of onset of depression?

a. Depression does not begin in adolescence

b. Depression can start in childhood or adolescence.

c. Depression appears for the first time in middle-aged people.

d. Depression does not affect young children.

5. Which of the following, about sex differences in depression is TRUE?

a. Only women get depressed.

b. Clinical depression is more common in women than men.

c. Clinical depression is more common in men than women.

d. Only men get depressed.

6. Which of the following is FALSE about the relapse of clinical depression?

a. The number of previous episodes of clinical depression increases the chances of subsequent episodes.

b. After the first episode of clinical depression, there is an increased risk of a second episode.

c. Maintenance treatment can reduce the chances of relapse.

d. After recovery, there is zero risk for recurrence.

7. Which of the following behavior is associated with poor outcome?

a. Taking antidepressant treatments regularly

b. Being involved in talk therapy (psychotherapy)

c. Staying sober

d. Stopping antidepressant medications if feeling well

8. What factors may trigger the onset of clinical depression?

a. Biological factors, such as genes

b. Psychological factors such as having marital problems

c. Social factors such as losing a job

d. All of the above

9. Depression may be triggered by all the following EXCEPT:

a. Prolonged severe grief over loved ones

b. Taking antidepressants

c. Certain medical conditions

d. The birth of a new baby

10. The following are indications of clinical depression EXCEPT:

a. Changes in sleep patterns

b. Poor concentration

c. Frequent crying for no obvious reasons

d. Occasional sadness

11. Which is NOT true about the differences between depression and a passing blue mood?

a. People with depression can "pull themselves together"

b. Depression can be much more disabling in day-to-day functioning.

c. Patients who are clinically depressed look sad.

d. Without treatment, symptoms of clinical depression can last for weeks, months, or years

12. All of the following are recognized symptoms of clinical depression EXCEPT:

a. Marked loss of interests.

b. Excessive sleep

c. Loss of energy

d. Good concentration

13. Which of the following is NOT a symptom of clinical depression?

a. Restlessness

b. Changes in appetite

c. Good decisions making

d. Lack of energy

14. All of the following are typical of patients suffering from clinical depression EXCEPT:

a. Negative thinking that can lead to self-defeating or suicidal behavior

b. Mental fatigue and the inability to solve complicated problems

c. Marked forgetfulness

d. Normal memory

15. Which is NOT a common symptom of clinical depression?

a. Poor motivation

b. Normal energy

c. Guilty thoughts

d. Fatigue

16. Which of the following statements about the speed of response to the treatment with antidepressants is FALSE?

a. Symptoms improve immediately after treatment is begun.

b. Many antidepressants may take several weeks to start to work.

c. It is important to continue taking medication even if there is initial improvement.

d. Not all symptoms respond to antidepressants at the same rate.

17. If medication does not improve depressive symptoms, one should:

a. Stop taking all medication.

b. Talk to a health care professional.

c. Double the pills.

d. Ask friends about what to do.

18. Which is NOT a recognized treatment for clinical depression?

a. Medication

b. Talk therapy.

c. Light therapy (photo-therapy).

d. Kiekie therapy

19. Which is NOT a common side effect antidepressant drugs?

a. Upset stomach

b. Sleep disturbances

c. Sexual side-effects (e.g. problems with sexual desire or orgasm)

d. Feelings of depression

20. Which is FALSE about the effectiveness of antidepressant medications?

a. About 30-40% of patients do not respond to the initial treatment.

b. Moderate symptom improvement may take few weeks to be achieved in those who will respond.

c. Using more than one antidepressant may be necessary for some patients.

d. Recovery of symptom can be achieved in all depressed patients

21. What should one do if one's first antidepressant medication fails?

a. Consult one's doctors

b. Take sleeping pills

c. Drink more alcohol

d. Use magnetic therapy

22. Which is FALSE about Electric Convulsive Therapy (ECT) for treating clinical depression?

a. It is proved to be effective.

b. It is a safe method.

c. It is no longer used for treating depression.

d. It is given under general anesthesia.

23. If one feels better during the course of treatment, one should

a. Stop taking antidepressant medication.

b. Discuss the course of antidepressants treatment with doctor.

c. Reduce the antidepressant dose by half.

d. Start a course of herbal treatment.

24. Which is NOT a common occurrence during treatment with antidepressants?

a. Gaining weight

b. Severe continuous headaches

c. Feeling sleepy

d. Sweating

25. Which is FALSE about the response to treatment with antidepressants?

a. Up to 80% of people with depression do get better with the right medication.

b. Most people with depression need to be treated for at least six to nine months to prevent relapse.

c. For some people, it is necessary to stay on medication for long-term maintenance therapy.

d. If the acute depressive symptoms are relieved, the patient should stop antidepressants.

26. Which is FALSE about selecting the right antidepressant for someone with depression?

a. There are no available laboratory tests to guide doctors' choices for treating clinical depression.

b. Different people have different responses to antidepressants.

c. Doctors can tailor antidepressants to suit the symptoms of individual patients.

d. Doctors can always tell beforehand how a person is going to respond to the medication they prescribe.

27. Psychotherapy can help many people with depression. Which of the following statements about psychotherapy is FALSE?

a. Both individual and group talk therapy provides an opportunity to express and discuss thoughts and feelings with the therapist.

b. Therapy may help to resolve life issues that may contribute to depression.

c. All depressed individuals benefit from psychotherapy.

d. In psychotherapy, negative, and self-defeating thoughts can be replaced by more positive, realistic thoughts.

## Pre-publication history

The pre-publication history for this paper can be accessed here:


